# Notes and News

**Published:** 1893-05-20

**Authors:** 


### Notes and News.

COLLECTED AND COMMUNICATED.

The Paris Municipal School of Physics and Chemistry has recently opened a chemical laboratory for students in their fourth year, to which not only former pupils of the school, but strangers to it also, are admitted.

There have been a good many cases of typhus fever in France lately, and in the districts where they have occurred the origin of the malady has been traced to strangers arriving from other parts. In consequence, an official order has been given to disinfect the third-class carriages on the Northern Railway daily. The Medical Society has issued a circular to all doctors, asking them to furnish a report on any cases that may come under their notice.

The report for the year 1892, read at the last annual meeting of the British and Foreign Sailors' Society, is a good one. The Society has now 91 stations, 76 institutes, rests, reading rooms, &c., and 138 agents. The Victoria and Albert Memorial Fund for the Assistance of Aged Missionaries now amounts to £3,350. During the past year 332 free floating libraries have been issued to ships in London and Gravesend, a boon for which alone the sailor owes the Society a great debt of thanks. Efforts are being made to complete the Albert Victor Memorial Fund, with the object of establishing a Sailors' Rest in the West India Dock Road.

Our usually accurate contemporary, the *New York Medical Record*, supplies us with news which if read by some of Sir Andrew Clark's numerous and influential patients may cause genuine alarm. We read that Sir Andrew Clark has resigned his post at St. Bartholomew's Hospital, and retired from private practice. Sir Andrew has not, nor has he ever had, any connection with St. Bartholomew's, and he is certainly not likely to be spared yet from his valuable labours. The gentleman referred to in the paragraph is, of course, Dr. Andrew, who recently resigned the office of Senior Physician, after many years' excellent work at St. Bartholomew's Hospital.

It would be useful to many if Lady Brassey's Home at Bexhill were more widely known. The situation is an excellent one for invalids, and the Home most comfortably furnished. Children from hospitals with unhealed wounds are admitted, and the Lady Superintendent has had much experience in nursing.

The returns of the Birmingham Hospital Saturday Fund this year show a gratifying increase, the collection amounting to £9,015, as against £8,739 collected in 189[illegible]. It is to be noted that the number of contributors shows a decrease, a point that rather goes towards proving that interest in hospital matters can be increased where it is once generated, but that it is not to be roused to an unlimited degree in the public.

Mr. Asquith brought forward a Bill in the House of Commons last week to abolish the cumulative penalties for infringing the vaccination law. It appears that all those who sat on the Royal Commission on Vaccination are in favour of this action, strange as it may seem. Statistics such as were revealed at Leicester in the immediate past on the occasion of the inquiry of the Sanitary Committee of the Town Council on the outbreak of small-pox in that town in April, and such as have been revealed by numbers of investigations elsewhere and on the Continent as to the mildness of the attack where vaccination has been effected, fail to convince opponents to the system. The opposition has been increasing, and where evasion has been particularly successful there the disease has in more than one instance gained a stronghold also. Cases of small-pox are uncomfortably numerous in the metropolis at the present time, and the "ill-wind" might be made to blow some good if the enemies to vaccination were to follow up the history of some hundred of cases for themselves.

The Princess Christian recently presented the certificates and badges of the St. John's Ambulance Association to members of the class held at Paddington Station. A knowledge of first aid to the wounded is eminently suitable to employés on all railways. A necessity to render help to those injured on the line may occur to officials at any moment, and it is a blessing indeed to the sufferers if the help rendered is in some degree intelligent and skilful. A contemporary has thought fit to sneer at the young lady pupils at ambulance classes, remarking that through such channels knowledge is not likely to be advanced. For our part, we hold that, however dangerous a little knowledge may be, a great deal of ignorance is worse. Those with practical acquaintance of the sick-room invariably pass through an exasperating initiation into the hopeless ignorance possessed by the majority. Lessons in common sense can never be thrown away, and those who sneer at well-meaning efforts to be of more use in the world incur a grave responsibility. Far better is it to utilise all means, however humble, towards a good end. In Germany Herr Jean Bungartz has succeeded in training dogs in the service of the Red Cross Society. These dogs have been exhibited before the Prussian War Ministry. They not only carry messages from one ambulance station to another, but seek and find the wounded on the battlefield. At the opening of the Imperial Institute the good services of the Ambulance Service were recognised on all sides, and their constant occupation showed how necessary an adjunct to a crowd an Ambulance Service is.

